# Emerging Roles of Claudins in Human Cancer

**DOI:** 10.3390/ijms140918148

**Published:** 2013-09-04

**Authors:** Mi Jeong Kwon

**Affiliations:** 1 College of Pharmacy, Kyungpook National University, 80 Daehak-ro, Buk-gu, Daegu 702-701, Korea; E-Mail: mjkwon94@knu.ac.kr; Tel.: +82-53-950-8581; Fax: +82-53-950-8557; 2 Research Institute of Pharmaceutical Sciences, College of Pharmacy, Kyungpook National University, 80 Daehak-ro, Buk-gu, Daegu 702-701, Korea

**Keywords:** claudin, cancer, epithelial to mesenchymal transition (EMT), cancer stem cells or tumor-initiating cells (CSC/TICs), chemoresistance, tumor recurrence, epigenetic regulation

## Abstract

Claudins are major integral membrane proteins of tight junctions. Altered expression of several claudin proteins, in particular claudin-1, -3, -4 and -7, has been linked to the development of various cancers. Although their dysregulation in cancer suggests that claudins play a role in tumorigenesis, the exact underlying mechanism remains unclear. The involvement of claudins in tumor progression was suggested by their important role in the migration, invasion and metastasis of cancer cells in a tissue-dependent manner. Recent studies have shown that they play a role in epithelial to mesenchymal transition (EMT), the formation of cancer stem cells or tumor-initiating cells (CSCs/TICs), and chemoresistance, suggesting that claudins are promising targets for the treatment of chemoresistant and recurrent tumors. A recently identified claudin-low breast cancer subtype that is characterized by the enrichment of EMT and stem cell-like features is significantly associated with disease recurrence, underscoring the importance of claudins as predictors of tumor recurrence. The critical role of epigenetic mechanisms in the regulation of claudin expression indicates the possible application of epigenetic therapy to target claudins. A better understanding of the emerging role of claudins in CSC/TICs and chemoresistance may help to develop therapies against recurrent cancers.

## 1. Introduction

Claudins are transmembrane proteins and important components of tight junctions (TJs), which are central for the regulation of paracellular permeability and the maintenance of epithelial cell polarity [1,2]. Their expression is altered in various cancers compared to normal tissues, and claudin-1, -3, -4 and -7 are among the most frequently dysregulated members of the claudin family [1,3]. The downregulation of several claudins in cancer is consistent with the disruption of TJs during tumorigenesis. However, claudin overexpression, particularly that of claudin-3 and claudin-4, has been reported in various cancers. The dysregulated expression of claudins in cancer occurs at both transcriptional and post-transcriptional levels [3]. Recently, accumulating evidence indicates that epigenetic mechanisms including DNA methylation, histone modification or microRNAs (miRNAs) are crucial for the regulation of claudin expression in addition to the previously reported transcriptional regulation by transcription factors [4–7]. Claudins can play a cancer-promoting or tumor suppressor role in a tissue-dependent manner, and their expression is associated with prognosis in several cancers, suggesting their utility as prognostic factors as well as therapeutic targets [1,3].

One of the main challenges in cancer therapy is overcoming chemoresistance and recurrence after initial treatment, which are partly responsible for the high mortality of several cancers. Recent studies have demonstrated the heterogeneity of tumors and suggested that a small population of cells with self-renewal potential—termed cancer stem cells or tumor-initiating cells (CSC/TICs)—play a crucial role in chemoresistance and recurrence, as well as in tumor formation [8]. This population of cells can be generated from differentiated cells (non-CSC/TICs) through epithelial to mesenchymal transition (EMT) [9]. In light of these observations, the existence of an axis between EMT, CSC/TICs and drug resistance was proposed [9].

Recently, a new claudin-low molecular subtype of breast cancer was identified that is characterized by low expression of tight junction and adherens proteins, including claudin-3, -4 and -7, and E-cadherin (CDH1) [10], and enriched in stem-like and EMT features [11,12]. In accordance with the role of EMT and CSC/TICs in cancer, this subtype is associated with poor prognosis of patients with high-grade invasive ductal breast carcinoma [13]. Furthermore, a growing number of studies suggest that claudins are involved in the regulation of CSC/TICs and chemoresistance [14–18]. These observations indicate that claudins may contribute to drug resistance and tumor recurrence through a mechanism involving EMT and CSC/TICs.

This review summarizes current knowledge on the dysregulation of claudins in human cancer and the regulatory mechanisms involved, focusing on epigenetic mechanisms whose importance in the regulation of claudin expression has been supported by recent growing evidence. In addition, the involvement of claudins in tumor progression, in particular their emerging roles in EMT and the formation of CSC/TICs, and the link between claudins, CSC/TICs and drug resistance or tumor recurrence are described. Finally, therapeutic strategies targeting claudins in cancer treatment are briefly discussed. A better understanding of the role of claudins in the regulation of CSC/TICs and chemoresistance may be of value for the design of therapeutic strategies for the treatment of recurrent tumors.

## 2. Tight Junctions

Tight junctions (TJs), together with adherens junctions and desmosomes, form the apical junctional complex that mediates cell-cell adhesion in epithelial and endothelial cells [19]. While adherens junctions and desmosomes mainly contribute to mechanical adhesion between adjacent cells, TJs are involved in several cellular functions. They play a critical role in the maintenance of cell polarity by forming a boundary between apical and basolateral membranes, and they control paracellular ion flux [19]. In addition, their involvement in various signaling pathways indicates that they function in the regulation of cell proliferation, gene expression and differentiation [19–21].

TJs are composed of integral transmembrane and peripheral membrane proteins involved in complex protein-protein interactions [19,21]. The integral transmembrane proteins include occludins, claudins, junctional adhesion molecules (JAMs), which contain a single transmembrane domain, and the single pass membrane proteins Crumbs 3 (CRB3) and coxackievirus and adenovirus receptor (CAR) [19,21]. Tricellulin, a four transmembrane domain protein, was recently identified as a novel integral protein [22]. Peripheral proteins located in cytoplasmic regions include zonula occludens (ZOs) and other PDZ domain-containing proteins, as well as regulatory and signaling proteins that link transmembrane TJ proteins to the cytoskeleton and regulate various signaling pathways [19,20].

Various associated proteins regulate TJ formation and cell polarization by controlling the transcription and localization of other TJ proteins. Two signaling complexes, the CRB3/protein associated with Lin seven 1 (PALS1)/PALS1-associated tight junction protein (PATJ) complex and the cdc42-interacting partitioning defective 3 (Par3)/partitioning defective 6 (Par6)/atypical protein kinase C (aPKC) complex, are important in the regulation of junctional polarity and assembly [21]. In addition to these complexes, scribble complex is also known to regulate cellular polarity and TJ formation in mammalian epithelial cells [23]. Interestingly, a recent study [24] reported that cell polarity regulator scribble regulates TAZ, which is a downstream effector of the hippo pathway and is required for self-renewal and tumorigenic potential of breast CSC/TICs, suggesting a link between cell polarity and hippo pathway in breast cancer cells. This study indicated that EMT-induced scribble delocalization from the cell membrane promotes the acquisition of breast CSC/TIC features by activating TAZ. Rab 13, a member of the small GTPase Rab family of proteins, also plays a role in TJ assembly by regulating the localization of TJ proteins including claudin-1 and ZO-1 to the plasma membrane and the protein kinase A (PKA)-dependent phosphorylation of proteins necessary for TJ barrier function [19].

In addition, TJ proteins are involved in cell proliferation and gene expression by modulating signaling cascades, or by sequestrating transcription factors and cell cycle regulators [20,21]. Occludin is linked to Raf, an effector of Ras signaling, and is also connected to Rho A signaling [21]. Occludin is involved in transforming growth factor-β (TGF-β)-induced EMT, which requires a loss of cell polarity and disruption of the TJ barrier [19,21]. The ZO-1-associated nucleic acid-binding protein (ZONAB), which is localized in both TJs and nuclei, is a Y-box transcription factor protein that regulates gene transcription and cell proliferation through interaction with the TJ protein ZO-1, the regulator of cell proliferation cell division kinase 4 (CDK4), or with Ras-like GTPase (RalA) proteins [21]. Generally, the assembly of TJs suppresses cell proliferation by inhibiting several proliferation promoting pathways such as Raf-1 signaling and the ZONAB-CDK4 pathway [20].

TJ-associated proteins such as ZO-2, membrane-associated guanylate kinase with inverted domain structure 1 (MAGI-1), and multi-PDZ domain protein 1 (MUPP1) inactivate viral oncogenes, and the interaction between the tumor suppressor PTEN and TJ adaptors—including MAGI-2 and MAGI-3—inhibits protein kinase B/Akt-induced cell invasiveness, suggesting that these TJ proteins play a role in carcinogenesis [19,20]. Changes in the activity of the ZO-2 interacting transcription factor AP-1 are associated with certain cancers, suggesting that TJ-associated signaling is dysregulated in cancer cells [19,25]. The regulation of the expression of the proto-oncogene ErbB2 by ZONAB [19,20] and alterations in the expression of TJ-associated proteins during tumorigenesis further support the possibility that these proteins are involved in tumorigenesis. ZO-1 and ZO-2 expression is altered in some cancers, and ZO-1 expression is associated with the prognosis of breast cancer patients [25]. However, whether these changes can induce tumorigenesis or if they are consequences of tumorigenesis remains unclear. Thus, further studies are needed to determine the role of TJ proteins in carcinogenesis.

## 3. Claudins

Claudins are major integral membrane proteins of TJs and include 24 family members in mammals [2]. In humans, 23 of the 24 claudin genes (the exception is *CLDN13*) have been identified (Table 1), while all 24 members have been detected in mice and rats [2]. *CLDN* genes have few introns and are typically small, *i.e.*, several kilobases (kbs). The human genome contains pairs of *CLDN* genes that have similar sequences and are located in close proximity, such as *CLDN6* and *CLDN9* on chromosome 16, *CLDN22* and *CLDN24* on chromosome 4, *CLDN8* and *CLDN17* on chromosome 21, and *CLDN3* and *CLDN4* on chromosome 7 (Table 1). This suggests that some claudin genes were generated by gene duplication, and that adjacent genes may be coordinately regulated [2]. Phylogenetic tree analyses of human claudin proteins also showed sequence similarities between some claudins, such as claudin-22 and claudin-24, claudin-6 and claudin-9, and claudin-3 and claudin-4, whereas other claudins show relatively distant relationships [2].

Most claudin proteins are within the 20–34 kDa size range (Table 1) and are reported to have four transmembrane helices with amino- and carboxyl-terminal tails extending into the cytoplasm [2,26]. In addition, claudin proteins have two extracellular loops; the first extracellular loop contains charged amino acids and plays a crucial role in paracellular ion selectivity [26]. The carboxy-terminal tails of claudins, which mostly differ in size and sequence between different claudin proteins, contain a PDZ-domain-binding motif that allows claudins to interact directly with cytoplasmic TJ-associated proteins such as ZO-1, ZO-2, ZO-3, and MUPP1. Moreover, this tail region is the site of post-translational modifications such as phosphorylation, which can affect the localization and functions of claudins. Phosphorylation of claudin-1 by mitogen-activated protein kinase (MAPK) [27] or protein kinase C (PKC) [28], and cyclic AMP (cAMP)-induced phosphorylation of claudin-5 [29] promote the barrier function of TJs. By contrast, PKA-mediated phosphorylation of claudin-16 increases Mg^2+^ transport [30]. Other proteins such as mutant WNK lysine-deficient protein kinase 4 (WNK4) also increase paracellular permeability by phosphorylating claudins [31].

The expression pattern of claudins varies among tissue types, and most tissues or cell types express multiple claudins [32,33]. Such multiple combinations of claudin expression contribute to the formation of TJs through their homotypic or heterotypic interactions, or their interaction with other TJ proteins [32]. Claudins play a crucial role in the regulation of the selectivity of paracellular permeability, with claudin-2 and claudin-15 known to function in cation channels/pores, whereas claudin-4, -7 and -10a contribute to the function of anion channels/pores [22]. Claudin overexpression in several cell lines affects transepithelial resistance (TER) and the permeability to different ions in a claudin-specific manner. Claudin-1, -4, -5 and -7 increase TER, whereas claudin-2 and claudin-10 decrease TER in cultured epithelial cells [22]. Moreover, claudin-4 overexpression alters Na^+^ permeability without significant effect on Cl^−^ permeability in Madin-Darby canine kidney (MDCK) cells [34].

Mutations in claudin genes have been linked to several human diseases. Sclerosing cholangitis and ichthyosis are associated with *CLDN1* mutation, and hypomagnesemia and hypercalcinuria have been linked to mutations in *CLDN16* and *CLDN19* [22]. Claudin-3 and claudin-4 are receptors for the *Clostridium perfringens* enterotoxin (CPE), while claudin-1, -6 and -9 are co-receptors for the hepatitis C virus (HCV).

## 4. Dysregulation of Claudins in Human Cancer

### 4.1. Claudin Expression in Human Cancers

Altered expression of several claudin proteins, in particular claudin-1, -3, -4 and -7, has been detected in various cancers (Table 2) [1,3]. Consistent with the disruption of TJs during tumorigenesis [1], certain claudins including claudin-1 and claudin-7 are downregulated in invasive breast, prostate, and esophageal cancers (Table 2). On the other hand, the upregulation of claudins, particularly claudin-3 and claudin-4, has also been associated with tumorigenesis. Claudin-3 and claudin-4 are highly overexpressed in ovarian cancer including serous carcinoma compared to normal ovarian tissues, and their expression is also upregulated in several other malignancies, including breast, gastric, pancreatic, prostate and uterine cancers (Table 2). It is important to note that the upregulation of claudin-3 and claudin-4 expression in ovarian cancer is based on the hypothesis that ovarian cancer arises from normal ovarian surface epithelium. However, recent studies have shown that most ovarian high-grade serous carcinomas originate from the fallopian tube rather than the ovarian surface epithelium [35–38]. In this context, the expression of claudins in serous ovarian carcinoma should be compared to that in the fallopian tube.

Recent studies on claudins in breast cancer showed that claudin expression may be distinct within specific subtypes of breast cancer, such as claudin-4, whose expression is downregulated compared to normal mammary epithelial cells in grade 1 ductal carcinoma of the breast [39], whereas it is significantly upregulated in basal-like breast cancer [40]. The expression of claudin-1, -3 and -4 is higher in the intestinal type of gastric adenocarcinoma than in the diffuse type of gastric cancer [41]. A recent comprehensive analysis of the expression of claudins-1, -3, -4, -7 and -8 in high-grade invasive breast cancer, including several molecular subtypes, demonstrated the differential expression of claudins according to molecular subtype, showing increased claudin-7 and -8 in luminal tumors (estrogen positive) and increased claudin-1 and-4 in basal-like tumors [13].

### 4.2. Regulation of Claudin Expression in Human Cancers

The expression of claudins can be regulated by multiple mechanisms [3]. First, claudin expression can be regulated at the transcriptional level by transcription factors. Snail, a transcriptional repressor that plays a crucial role in EMT, represses the gene expression of claudins-1, -3, -4 and 7 and E-cadherin in mouse epithelial cells by directly binding to their promoter regions [70]. In human cell lines, the transcription factors Slug and Snail bind to the E-box in the promoter of human claudin-1, inhibiting its expression [71]. This study also showed the inverse correlation between the low expression level of claudin-1 and the high expression of these two transcription factors in human invasive breast cancer. Conversely, transcription factor RUNX3, which is a gastric tumor suppressor, upregulates claudin-1 expression by binding to the promoter region of *CLDN1* in gastric epithelial cells [72]. In colon cancer cells, caudal homeobox proteins (Cdx1 & Cdx2) and GATA4 in cooperation with the Wnt pathway are involved in claudin-1 promoter activation [73].

Recent studies have shown the importance of epigenetic mechanism in the transcriptional regulation of *CLDN* expression. DNA hypermethylation is associated with the downregulation of *CLDN11* in gastric cancer cells [53] and *CLDN7* in breast cancer cells [45]. The association between promoter DNA hypermethylation and low expression of claudin-1 was also reported in estrogen receptor-positive breast cancer [74]. By contrast, claudin-4 overexpression in ovarian cancer is associated with DNA hypomethylation, whereas gene amplification is not related to claudin-4 expression [75]. In addition to DNA methylation, our group reported that loss of repressive histone methylations, including H3K27me3 and H4K20me3, is also associated with the overexpression of claudin-3 and claudin-4 in ovarian cancer [6] and claudin-4 in gastric cancer [7]. Our study in ovarian cancer [6] suggested that epigenetic derepression in addition to the well-known epigenetic inactivation of tumor suppressor genes may be a possible mechanism underlying the activation of cancer-associated genes. Another recently reported epigenetic mechanism is the regulation of *CLDN* expression by miRNAs. For example, claudin-1 mRNA and protein expression is downregulated by miR-155 in ovarian CSC/TICs [16].

Studies have shown that transcription factors and epigenetic modifications cooperate in the transcriptional regulation of claudin levels. The Sp1 transcription factor is crucial for *CLDN3* and *CLDN4* promoter activity, and the epigenetic status, including DNA methylation and histone H3 acetylation, in the critical region containing the Sp1 binding site also plays a role in the regulation of *CLDN3* and *CLDN4* expression in ovarian cancer cells [4,5]. DNA hypomethylation and the transcription factor CREB are related to the transcriptional upregulation of *CLDN18* splice variant 2 [76]. Claudin-18 mRNA and protein levels are upregulated by treatment with 12-*O*-tetradecanoylphorbol 13-acetate (TPA), a PKC activator, and this upregulation is further enhanced by treatment with DNA demethylating agents in human pancreatic cancer cells [77].

Claudin expression may also be regulated at the post-transcriptional level. Claudin-1 expression was shown to be regulated through the modulation of mRNA stability in colon cancer cells [78], and further analysis by the same group showed that histone deacetylase (HDAC)-dependent regulation of claudin-1 mRNA stability is mediated by the binding of Hu antigen R and Tristetraprolin to the 3′ UTR of claudin-1 mRNA [79]. On the other hand, claudin-1 expression is increased by PKC in melanoma cells and increased claudin-1 levels contribute to melanoma cell invasion and motility [55]. Claudin-7 expression is negatively regulated by Wnt/Tcf signaling via Sox-7 in colorectal cancer cells [80]. Claudin-4 expression is downregulated by TGF-β, whereas Ras signaling positively regulates claudin-4 expression in pancreatic cancer cells [81]. Furthermore, growth factors, such as the epidermal growth factor, can regulate claudin expression along with TER [82]. Moreover, nonsteroidal anti-inflammatory drugs such as aspirin significantly decrease claudin-7 expression in association with p38 MAPK activation in gastric epithelial cancer cells [83].

## 5. Role of Claudins in Human Cancer

### 5.1. Claudins in Tumor Progression

The altered expression of claudins in various cancers plays different roles in a tissue-specific manner (Table 3). Epithelial tumor cells lose TJ function, leading to the loss of cell polarity and impairment of epithelial integrity during tumorigenesis [1,84]. Accordingly, loss of claudin expression was assumed to contribute to tumor progression in association with the loss of cell adhesion [1,3]. However, a number of studies have shown that increased expression of claudins may promote tumor progression through its positive effect on cell migration, invasion and metastasis (Table 3). In addition to such aberrant expression, the mislocalization of claudin proteins may contribute to their role in tumorigenesis. For example, delocalization of claudin-1 and claudin-4 from TJs in bladder tumors [85] and the effects of altered localization of claudin-7 on the invasiveness of esophageal carcinoma [84] have been reported. Additionally, the function of claudins is regulated by post-translational modifications such as phosphorylation. Phosphorylation of serine and/or threonine sites in the carboxyl-terminal domains of claudins by PKA or PKC can influence their role in cancer cells, as in ovarian cancer cells, where it increases paracellular permeability [86,87]. Several signaling pathways are involved in the roles of claudins in tumorigenesis [3]. The detailed function of some claudins reporting their significant roles in various cancers will be described in the next sections.

#### 5.1.1. Claudin-1

Claudin-1 is one of the most dysregulated claudins in human cancers, and its crucial role in various cancers has been described (Table 3). Claudin-1 can function as a cancer-promoting and tumor suppressor factor depending on cancer type.

The cancer-promoting role of claudin-1 via its effect on invasion or motility of cancer cells has been described in various cancers. Claudin-1 significantly increases xenograft tumor growth and metastatic behavior in athymic mice through its effects on E-cadherin expression and β-catenin/Tcf signaling in colon carcinoma [48]. This study also showed that claudin-1 expression increases in colon cancer, in particular in metastastic tissues with mislocalization from the cell membrane to the cell nucleus and cytoplasm. Claudin-1 overexpressing colon cancer cells formed more colonies in soft agar than did control cells and increased the activity of matrix metalloproteinases (MMPs). In contrast, inhibition of claudin-1 expression significantly decreased the anchorage-independent growth and invasion of metastatic colon cancer cells with a significant decrease in MMP-9 activity. In oral squamous cell carcinoma cells, claudin-1 promotes invasion by upregulating the activity of MMP [92]. Similarly, claudin-1 expression induces MMP-2 activation, resulting in increased cell invasion and motility in melanoma cells [55]. In this study, claudin-1 expression was increased at the mRNA and protein levels by PKC activation, while the inhibition of PKC signaling decreased claudin-1 expression. In human liver cells, claudin-1 promotes invasive behavior by activating the c-Abl-PKC signaling pathway [90]. Recently, Suh *et al*. [91] in the same group further revealed that the induction of EMT by claudin-1 requires the activation of the c-Abl-Ras-Raf-1/ERK1/2 signaling pathway in human liver cells, supporting the importance of c-Abl signaling in the claudin-1-induced acquisition of a malignant phenotype. Claudin-1 knockdown in basal-like breast cancer cells decreases cell migration by affecting the expression of genes involved in EMT [88].

On the other hand, claudin-1 has an anti-apoptotic effect in tamoxifen-treated human breast cancer MCF-7 cells [89].

Conversely, the tumor suppressive activity of claudin-1 was reported in gastric cancer [72] and lung cancer [93]. Knockdown of claudin-1 in gastric cancer cells increases *in vivo* tumorigenicity [72], and claudin-1 overexpression suppresses metastasis as well as cell migration and invasion of lung cancer cells [93].

#### 5.1.2. Claudin-3 and Claudin-4

Because of the frequent dysregulation of claudin-3 and claudin-4 in ovarian cancer cells, the role of these claudins has mainly been reported in ovarian cancer (Table 3). In particular, overexpression of claudin-3 or claudin-4 in ovarian cancer is based on the hypothesis that ovarian cancers originate from normal ovarian surface epithelial cells, which do not express claudin-3 or claudin-4. Therefore, the overexpression of claudin-3 or claudin-4 has been shown to promote the progression of ovarian cancer. Forced expression of claudin-3 and claudin-4 in ovarian epithelial cells increases invasive behavior by inducing the activation of MMP [94]. *CLDN3* small interfering RNA (siRNA) inhibits tumor growth and metastasis in mouse and human ovarian tumor xenografts, further supporting the cancer-promoting role of claudin-3 [95]. Claudin-4 promotes the production of factors that stimulate angiogenesis both *in vitro* and *in vivo*, suggesting its pro-angiogenic role in ovarian cancer [97]. A recent study showed that claudin-4 promotes the motility of breast or ovarian cancer cells through interactions of its second extracellular loop with extracellular matrix proteins [104].

However, the opposite function of claudin-3 and claudin-4 in ovarian cancer was recently reported by Shang *et al.* [64]. In this study, knockdown of claudin-3 and claudin-4 enhanced *in vivo* tumor growth and lung metastasis, whereas a significant growth increase was not observed *in vitro*. TJ formation and cell adhesion is impaired in claudin-3 and claudin-4 knockdown ovarian cancer cells. Loss of claudin-3 or claudin-4 expression increases *in vitro* cell migration and invasion in line with their effect on enhancing metastasis *in vivo*. Moreover, this phenomenon is associated with downregulation of E-cadherin and activation of β-catenin signaling. Based on these results, this study concluded that both claudin-3 and claudin-4 inhibit tumor growth and metastatic potential *in vivo* through the maintenance of E-cadherin expression and suppression of β-catenin signaling, suggesting a tumor suppressive role for claudin-3 and claudin-4 in ovarian cancer. This study also examined the expression of claudin-3 and claudin-4 in the distal fallopian tube and in tumors from the same patients in six cases of serous ovarian cancer, and high expression of these claudins in both sites was observed in all six patients, suggesting that claudin-3 and claudin-4 are normally expressed in the fallopian tube, and downregulation of claudin-3 or claudin-4 in ovarian cancer promotes tumor growth and metastatic behavior *in vivo*.

This relationship was further reinforced by results from the same group. Lin *et al*. investigated the effect of the knockdown of these proteins on the expression of EMT markers and showed that inhibition of claudin-3 and claudin-4 promotes EMT in ovarian cancer cells through the downregulation of E-cadherin, upregulation of Twist, and activation of the PI3K pathway [96]. These results are consistent with an increasing body of evidence suggesting that high-grade serous ovarian carcinomas arise from the distal fallopian tubes rather than the ovarian surface epithelium [36–38]. In contrast to the roles of claudin-3 and claudin-4 in ovarian cancer cells, claudin-1 promotes EMT in human liver cells, supporting the notion that the role of claudins in EMT is tissue-specific [67].

In light of these two recent studies, it may be postulated that ovarian cancers lacking the expression of these claudins show a more aggressive and metastatic behavior, which contradicts the previously accepted concept that claudin-3 or claudin-4 overexpression in ovarian carcinoma is related to a more malignant phenotype of ovarian cancer. However, these recent studies indicating the tumor suppressive role of claudin-3 or -4 in ovarian cancer cells are based on results obtained in only one or two cell lines. Furthermore, these findings are in conflict with the association of high claudin-3 expression with poor prognosis of ovarian cancer patients, including the shorter survival of patients previously reported by our group [105]. Therefore, further comprehensive studies are necessary to elucidate the exact role of claudin-3 and claudin-4 in ovarian cancer.

The association between the loss of claudin-3 and claudin-4 and the degree of malignancy of ovarian cancer is in line with the known disruption of TJ function during tumorigenesis, and may explain the relationship between the claudin-low subtype with low expression of claudin-3, -4 and -7 and E-cadherin and the aggressive phenotype of breast cancer [11,12].

Similarly, the tumor suppressive role of claudin-4 has been described in various cancers. Claudin-4 expression suppresses cell invasion and metastasis in pancreatic cancer [81]. Our group also showed that claudin-4 overexpression inhibits the migration and invasion of gastric cancer cells without affecting cell growth [7]. Evidences showing that low claudin-4 expression is linked to poor prognosis of patients with breast [106], esophageal [107], colon [108,109] and pancreatic cancers [110] suggest that claudin-4 expression is likely to play a tumor suppressive role in several cancers, although its role in ovarian cancer remains unclear.

The function of claudin-3 or claudin-4 is regulated by phosphorylation via kinases as well as by forced or knockdown expression. For example, phosphorylation of claudin-3 and claudin-4 by PKA [86] or PKC [87] increases paracellular permeability in ovarian cancer cells via mislocalization of claudins. As in ovarian cancer cells, PKCα activation results in the mislocalization of claudin-4 along with decreased tight junction barrier integrity in human pancreatic cancer cells [111].

#### 5.1.3. Claudin-6

Despite several reports describing claudin-6 expression in multiple human cancers such as rhabdoid tumors [112], breast cancer [113] and gastric cancer [114], the function of claudin-6 in cancer cells has not been analyzed in detail. One study showed that decreased expression of claudin-6 enhances anchorage-independent growth and promotes cellular invasiveness of breast cancer cells [99]. In another study [100], claudin-6 expression was associated with decreased anchorage-independent growth, invasion, and increased TER in breast carcinoma cells, suggesting a possible tumor suppressive role for claudin-6 in breast cancer. On the other hand, claudin-6 overexpression in the human gastric cancer cell line AGS increases its invasion, migration, and proliferation potentials [98].

A recent study suggested a novel role for claudin-6 as a receptor for CPE by showing that the CPE sensitivity of an ovarian cancer cell line that does not express claudin-3 and claudin-4 is decreased in response to claudin-6 knockdown, while ovarian cell lines resistant to the effects of CPE can be made sensitive through claudin-6 overexpression [115]. However, further studies are required to support the role of claudin-6 as CPE receptor.

#### 5.1.4. Claudin-7

Claudin-7 is another claudin that is frequently dysregulated in cancer, and several studies have reported its role in cancer. Knockdown of claudin-7 expression in esophageal squamous cell carcinoma cells induces loss of E-cadherin, along with increased cell growth and enhanced cell invasion [84]. Similarly, claudin-7 inhibits the migration and invasion of lung cancer cells through a mechanism involving the ERK/MAPK signaling pathway [102].

By contrast, studies have shown that claudin-7 may promote tumor progression. Claudin-7 overexpression in colorectal cancer cells disrupts cell polarization, enhances β-catenin/Tcf activity and cell proliferation, and thereby promotes tumor formation *in vivo* in xenograft mice injected with claudin-7 overexpressing colorectal cancer cells [80]. The EpCAM-claudin-7 complex rather than EpCAM itself was reported to promote *in vivo* tumor growth [116]. The migration and invasion of ovarian cancer cells is also enhanced by claudin-7 overexpression [101].

#### 5.1.5. Claudin-11

Claudin-11, also known as oligodendrocyte-specific protein (OSP), is a major component of central nervous system myelin and was shown to be highly regulated during development, suggesting its role in the growth and differentiation of oligodendrocytes [117]. However, its function in cancer is not well understood. The function of claudin-11 in cancer cells has been mostly associated with its tumor suppressor function. Reduced *CLDN11* expression is associated with increased invasiveness of gastric cancer cells [53]. Claudin-11 decreases the invasiveness of bladder cancer cells [103], and knockdown of claudin-11 in glioma stem cells promotes cell proliferation, supporting its tumor suppressor function [118].

### 5.2. Claudins in Cancer Stem Cells or Tumor-Initiating Cells

The recently reported involvement of CSC/TICs in tumor recurrence and drug resistance has generated much interest in elucidating the molecular mechanism regulating these cell populations [8]. Moreover, the EMT process has been involved in the generation of CSC/TICs [9]. Previous studies have shown that claudins are involved in the EMT process [91,96], and recent studies showing the enrichment of stem-like and EMT features in the claudin-low subtype of breast cancer provided new insight into the role of claudins in CSC/TICs [11,12].

The claudin-low subtype is a recently identified molecular subtype of human breast cancer characterized by low expression of tight junction and adherens genes including *CLDN3*, *CLDN4*, *CLDN7* and *CDH1* [10]. Most claudin-low tumors are triple-negative breast cancers (TNBCs) which lack the expression of estrogen receptor, progesterone receptor and epidermal growth factor receptor 2 (HER2) and they were found to be distinct from basal-like tumors [12]. However, it is possible that mesenchymal features of claudin-low tumors are derived from tumor-associated fibroblast or stromal contamination, not from tumor cells, and therefore further evaluation of this subtype is needed to rule out this possibility. Although this molecular subtype is not fully characterized at present and there are controversies surrounding the concept or presence of this subtype, a growing body of studies has suggested the molecular or clinical significance of the claudin-low subtype of breast cancer, as described in the following sections.

First, Hennessy *et al*. [11] compared the transcriptional profiling of metaplastic breast cancers (MBCs), which are an aggressive and chemoresistant subgroup of TNBCs, with other common breast cancer subtypes including luminal, HER2-enriched and basal-like cancers and identified that MBCs are the most related to the recently identified claudin-low breast tumors. This study also showed that MBCs and claudin-low subtype breast tumors have high levels of stem cell and EMT markers, and that the transcriptional features of these subtypes are enriched in CD44^+^/CD24^−/low^ breast CSC/TICs. Another study by Creighton *et al.* [17] showed that the CD44^+^/CD24^−/low^-mammosphere (MS) signature is primarily found in the claudin-low molecular subtype. Moreover, an increase in CD44^+^/CD24^−/low^-MS and claudin-low signatures was observed in post-treatment samples after chemo or hormone therapy, indicating that remaining breast tumors after conventional treatment are likely to be enriched in cell subpopulations with CSC/TIC features. Prat *et al*. [12] comprehensively analyzed the clinical and molecular characteristics of the claudin-low subtype of breast cancer in comparison to those of the other subtypes and showed that claudin-low breast tumors are correlated with poor prognosis and enriched in mesenchymal and mammary stem cell-like features. More recent study by Lehmann *et al*., [119] identified six TNBC subtypes by analyzing the gene expression profiles of 587 TNBC cases. Among the six subtypes of TNBC, the mesenchymal stem-like (MSL) subtype was found to display low expression of claudin-3, -4, and-7, consistent with claudin-low tumors, indicating that this subtype is in part composed of the claudin-low tumors. Collectively, these results may support the high association between low claudin expression and the features of mammary CSC/TICs.

Several recent studies showed the generation of breast CSC/TICs with claudin-low phenotypes. Asiedu *et al*. [120] indicated that cells with breast CSC/TIC phenotypes can be generated through TGF-β/TNF-α-mediated EMT in mouse mammary carcinoma cells. These cells showed enhanced *in vitro* self-renewal capacity and *in vivo* tumorigenicity as well as increased resistance to drug treatments consistent with the phenotype of CSC/TICs. Furthermore, this study revealed that TGF-β/TNF-α-derived breast CSCs/TICs show downregulated expression of genes encoding claudins-3, -4 and -7, luminal markers and cytokeratin 18, confirming the generation of claudin-low breast CSC/TICs through EMT. More recent studies confirmed that TGF-β can increase the number of CSC/TICs in breast cancer cell lines [121] and showed that EMT-inducing transcription factors in cooperation with active RAS are sufficient to drive the transformation of human mammalian epithelial cells into malignant cells with the features of claudin-low tumors in transgenic mice [122]. However, it is not yet demonstrated whether loss of claudin expression has a causative role in the generation of breast CSC/TICs via EMT or claudin-low subtype of breast cancer is just a secondary consequence of disruption of cell-cell junctions and loss of cell polarity during EMT independent of its effects on CSC/TICs. Further investigations are required to elucidate the role or functional significance of claudins in breast CSC/TICs.

The potential role of claudins in the regulation of CSC/TICs has also been reported in other cancers. First, knockdown of claudin-4 expression in ovarian cancer cells delayed spheroid formation, suggesting the involvement of claudin-4 in the regulation of spheroid formation [123]. In addition, microarray analysis comparing gene expression between CD133^+^CD117^+^ primary ovarian sphere cells and differentiated cells identified claudin-1 as one of the genes significantly upregulated in ovarian CSC/TICs, suggesting the possible role of claudin-1 in ovarian CSC/TICs [15]. Based on these findings, the same group showed that miR-155 downregulates claudin-1 expression at the mRNA and protein levels by targeting its mRNA in ovarian CSC/TICs [16]. This study also showed that miR-155 overexpression significantly suppresses the proliferation and invasion of ovarian CSC/TICs *in vitro* and the growth of ovarian CSC/TIC xenograft tumors *in vivo*. These results indicate that miR-155 suppresses the proliferation of ovarian CSC/TICs by downregulating claudin-1 expression and provide further evidence supporting the role of claudin-1 in ovarian CSC/TICs. Independent of its effect on CSC/TICs, overexpressed miR-155 in colorectal cancer cells promotes the migration and invasion of cells through the upregulation of claudin-1 expression [124].

Unlike other claudins, claudin-6 is expressed at higher levels in undifferentiated mouse stem cells than in non-stem cells [125] and plays an important role in the development of the mouse embryonic epithelium [126]. Claudin-6 is also highly expressed in human undifferentiated cells and is necessary for human pluripotent stem cell survival and self-renewal [127]. These findings support the crucial role of claudin-6 in the function of human pluripotent stem cells and suggest its value as a stem cell-specific marker and as a target for the elimination of undifferentiated cells from mixed cell populations. Given its role in the function of pluripotent stem cells, claudin-6 is likely to play a role in CSC/TICs. Therefore, further studies investigating the involvement of claudin-6 in CSC/TICs are necessary to understand its role in their regulation.

Claudin-11 significantly inhibits the growth of glioma stem-like cells [118], and the expression of claudin-11 is regulated by miR-1275, indicating the importance of epigenetic mechanism in the regulation of glioma stem-like cells.

The relevance of claudins in CSC/TICs is beginning to emerge, and further investigation aimed at elucidating their function in CSC/TICs may support the notion that claudins are promising targets for the regulation of CSC/TICs in the treatment of recurrent cancers.

### 5.3. Claudins in Chemoresistance

Resistance to chemotherapy is the main cause of treatment failure in many cancers [128]. Thus, identifying cancer chemoresistance-associated genes or pathways is critical for the successful treatment of cancer.

As the development of effective therapies against chemoresistant tumors is a high priority in ovarian cancer, several studies have focused on the identification of chemoresistance-associated genes or pathways. To identify proteins associated with cisplatin resistance in ovarian cancer, Stewart *et al*. [129] compared the expression of 1117 proteins between cisplatin-sensitive and -resistant ovarian cancer cells using quantitative proteomics technology and identified 121 proteins differentially expressed between the two cell lines. Of note, claudin-4 was overexpressed by 7.2-fold in cisplatin-resistant cells and was one of the most overexpressed proteins, suggesting its possible association with cisplatin resistance in ovarian cancer.

Consistent with this study, several lines of evidence support the association of high claudin-4 expression with chemoresistance of ovarian cancer. A recent study by a Japanese group [130] reported that ovarian cancer tissues from platinum-resistant patients have higher levels of claudin-4 expression than those from chemosensitive patients, and suppression of claudin-4 increased the sensitivity of ovarian cancer cells to cisplatin. Furthermore, higher claudin-4 gene expression was also observed in chemoresistant CD44^+^ ovarian CSC/TICs [131].

Another study showed that the expression of claudin-3 and claudin-4, which are CPE receptors, is significantly higher in chemotherapy-resistant/recurrent ovarian tumors than in chemotherapy-naive ovarian cancers, and that targeting these proteins by CPE-mediated therapy may be effective in killing chemoresistant/recurrent ovarian tumors *in vitro* and *in vivo* [132].

In addition to claudin-3 and claudin-4, claudin-7 expression is also involved in the response to platinum-based chemotherapy. Kim *et al*. [133] reported that high claudin-7 protein expression is significantly associated with shorter progression-free survival and poor sensitivity to platinum-based chemotherapy in ovarian cancer patients. In line with these clinical observations, claudin-7 knockdown was shown to increase the sensitivity of ovarian cancer cells to cisplatin treatment in this study. These findings were supported by a report showing that EpCAM-associated claudin-7 promotes drug resistance in a rat pancreatic cancer cell line [134].

Taken together, these results indicated that the expression of claudins, including claudin-3, -4 and -7, is higher in chemoresistant than in chemosensitive ovarian cancer cells and that their high expression is associated with increased resistance to chemotherapy in ovarian cancer, highlighting the fact that targeting these claudins using CPE or siRNA may increase the sensitivity of ovarian cancer cells to chemotherapy.

However, the opposite results on the association of these claudins with chemoresistance have been recently reported by a growing number of studies. Shang *et al*. [18] showed that knockdown of claudin-3 or claudin-4 increases the resistance to cisplatin in ovarian cancer cells *in vitro* and *in vivo*. Furthermore, these authors investigated the molecular mechanism by which claudin-3 or claudin-4 affect cisplatin sensitivity by assessing the changes in the expression of Cu transporters and chaperones based on previous studies showing their role in the sensitivity of ovarian cancer cells to cisplatin. The results showed that the effect of claudin-3 or claudin-4 on the sensitivity of ovarian cancer cells to cisplatin is mediated in part by the regulation of the expression of the copper and cisplatin influx transporter CTR1. CTR1 gene expression was reduced in *CLDN3* or *CLDN4* knockdown cells, and enforced CTR1 expression restored their sensitivity to cisplatin, indicating that CTR1 plays a role in cisplatin sensitivity in ovarian cancer cells.

Similarly, a recent study on human lung cancer cells showed that claudin-7 increases chemosensitivity to cisplatin by activating caspase pathways [135], which is in contrast to the study by Kim *et al*. [133] that showed an association between high claudin-7 expression and poor sensitivity to platinum-based chemotherapy in ovarian cancer. In this study, claudin-7-transfected cells showed a remarkable increase in the rate of cell apoptosis and significantly decreased cell viability. Cisplatin treatment increased the levels of cleaved caspases and PARP in claudin-7-transfected cells compared to non-transfected cells, indicating that increased chemosensitivity in claudin-7-expressed cells is related to the caspase pathway.

Emerging evidence on the association of CSC/TICs with chemoresistance suggests that the reduced levels of claudin-3, -4, or -7 expression in ovarian cancer cells is related to increased resistance to chemotherapy based on recent studies showing that reduced claudin-3 or claudin-4 promote EMT in ovarian cancer cells [96]. The association of low claudin expression with resistance to chemotherapy is supported by a recent study showing that subpopulations of CD44^+^/CD24^−/low^ breast cancer cells with high CSC/TIC potential and a claudin-low subtype are enriched in residual tumors after conventional chemotherapy, which is in line with the expectation of survival of CSC/TICs after treatment [17] and indicates that the claudin-low subtype of breast cancer is associated with increased resistance to chemotherapy.

In addition to the aforementioned claudins, including claudin-3, -4 and -7, accumulating evidences support the link between reduced claudins and increased resistance to chemotherapy of cancer cells. To identify specific signaling pathways associated with platinum resistance in ovarian cancer, Li *et al*. [136] performed an integrated analysis of global DNA methylation and gene expression in cisplatin-sensitive or -resistant ovarian cancer cells. A series of drug-resistant ovarian cancer cells were established by successive cisplatin treatments in the drug-sensitive A2780 ovarian cancer cell line. Promoter DNA methylation analyses showed an increase in the number of hypermethylated CpG islands and GI50 values (*i.e.*, dose necessary for 50% growth inhibition) with successive cisplatin treatment cycles, indicating the positive correlation between DNA methylation and the development of cisplatin resistance. Furthermore, pathway enrichment analysis identified several key biological pathways repressed by DNA hypermethylation or activated by DNA hypomethylation, and *CLDN11* was identified as one of the genes involved in pathways repressed by DNA hypermethylation during the development of cisplatin resistance.

Consistent with these results, reduced claudin-1 expression was associated with increased chemoresistance of epithelial cancer cells [137] in a study in which stable knockdown of keratin 8 and 18 increased cisplatin-induced apoptosis through claudin-1 upregulation, suggesting that increased claudin-1 expression may increase cisplatin sensitivity [137].

Taken together, these studies indicate that claudins contribute to drug resistance in cancer cells via their effects on drug transporters, apoptosis or CSC/TICs, although the exact underlying mechanism is not well understood. In addition, the differences in the relationship between claudins and chemoresistance according to cancer type and the reason behind the reported opposite results on the function of the same claudin remain unclear. Thus, further comprehensive studies are required to shed light on the relevance and role of claudins in chemoresistance.

**Figure 1:**
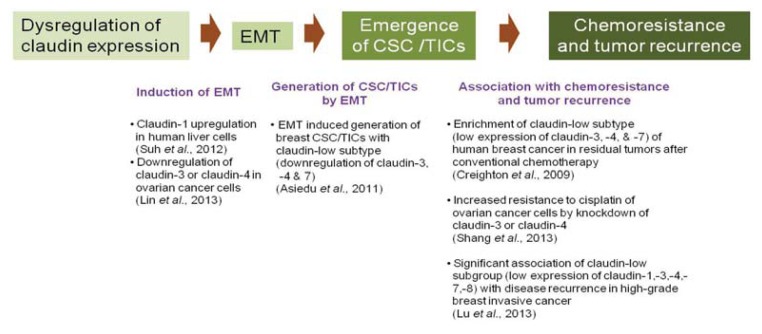
Proposed emerging role of claudins in epithelial to mesenchymal transition (EMT), cancer stem cells or tumor-initiating cells (CSC/TICs), and chemoresistance or recurrence.

In summary, the recent emerging roles of claudins in EMT, CSC/TICs and chemoresistance suggest the existence of a link between claudins, EMT, CSC/TICs and chemoresistance (Figure 1).

## 6. Claudins as Biomarkers and Therapeutic Targets

### 6.1. Prognostic Significance of Claudins in Cancer

The highly specific claudin expression patterns in human cancer tissues suggest that claudins may be useful biomarkers for the detection, diagnosis, and treatment of cancers. Among claudin proteins, claudin-3 and claudin-4 have been shown to be highly upregulated in ovarian carcinoma compared to normal ovarian surface epithelium in several studies [57,58,61,62]. Because of their consistent overexpression in ovarian cancer, claudin-3 and claudin-4 have been suggested as potentially useful biomarkers for the detection and diagnosis of ovarian cancer. Recently, claudin-4 was detected in the peripheral circulation of ovarian cancer patients, further supporting its usefulness as a biomarker [138].

Claudin expression has also been reported as a prognostic indicator as dysregulated claudin expression is associated with the prognosis of cancer patients (Table 4).

Low expression of claudin-1 is associated with poor patient prognosis in several cancers, including stage II colon cancer [139] and prostate cancer [65], and it is an independent predictor of tumor recurrence in both cancer types. Decreased claudin-1 expression is an indicator of the degree of malignancy of hepatocellular carcinoma as suggested by its correlation with dedifferentiation and portal invasion [54]. Similarly, decreased claudin-1 expression is positively correlated with the frequency of recurrence and shorter disease-free intervals in breast cancer [140]. Claudin-1 expression is upregulated in association with older age in women with basal-like breast cancer, suggesting the potential value of claudin-1 for the identification of specific groups of patients with basal-like breast cancer [88].

Similarly, the downregulation of claudin-2 protein expression is significantly associated with a high clinical stage of patients with breast cancer [43].

By contrast, ovarian serous adenocarcinoma patients with high claudin-3 expression show a significantly shorter survival than those with low claudin-3 expression, while claudin-4 expression is not associated with patient survival in this cancer [105]. Therefore, claudin-3 overexpression is an independent negative prognostic factor in ovarian serous carcinoma. Claudin-3 and claudin-4 overexpression in this study was determined by their non-detectable expression in normal ovarian surface epithelium based on the notion that ovarian cancer originates in the normal ovarian surface epithelium [105].

A study investigating the clinical significance of claudin-4 in breast cancer showed that high claudin-4 expression is associated with significantly shorter overall survival and recurrence-free survival, suggesting a relationship between high claudin-4 expression and poor outcomes of patients with breast cancer [142]. However, recent studies have shown that the distinct prognostic significance of claudin-3 or claudin-4 is dependent on the subtype of breast cancer. For instance, a Japanese group showed that the combination of claudin-4 and E-cadherin expression called CURIO accurately predicts relapse-free survival in breast cancer [106]. CURIO was shown to predict prognosis, especially in luminal A and triple-negative subtypes of breast cancer: high expression of CURIO is related to worse prognosis and low expression is associated with a better outcome. The distinctive prognostic significance of claudin-3 and claudin-4 in triple-negative and luminal types of breast cancer was analyzed in a recent report [141] in which positive expression of claudin-3 was associated with poor prognostic factors, whereas claudin-4 expression was related to better prognostic factors in TNBCs. Conversely, positive claudin-4 expression was associated with shorter disease-free survival and claudin-3 was related to longer disease-free survival in luminal types of breast cancer.

The relationship of claudin-4 expression with patient prognosis in gastric cancer is also controversial. One study [41] showed that strong claudin-4 expression is more frequently associated with the intestinal type than the diffuse type of gastric cancer, and high claudin-4 expression is significantly associated with shorter survival, while another study [147] showed that overall survival is decreased in patients with low claudin-4 expression. Our group recently reported that high membranous claudin-4 expression is related to better prognosis, while cytoplasmic claudin-4 expression has no significant relationship with patient prognosis [7]. Importantly, this study demonstrated that high membranous claudin-4 expression is an independent positive prognostic factor in gastric carcinoma.

Low expression of claudin-4 is related to poor prognosis in esophageal cancer [107], colon cancer [108], colorectal cancer [109], and pancreatic carcinoma [110].

Reduced expression of claudin-7 is associated with poor patient outcome in several cancers. Comparison of the expression of claudin-7 at the invasive front of the esophageal cancer with that in the corresponding metastastic lymph nodes showed significantly reduced claudin-7 expression in metastastic lymph nodes, indicating that claudin-7 may be a predictor of lymph node metastasis [50]. Loss of claudin-7 expression is significantly correlated with high histological grade of breast carcinoma, including ductal carcinoma *in situ* and invasive ductal carcinoma [45]. A recent study further supported the poor prognostic significance of claudin-7 expression in ductal invasive breast cancer by showing that claudin-7 expression is associated with a shorter time to recurrence [143]. In non-small cell lung cancer, survival is significantly poorer in patients with low claudin-7 expression than in those with high claudin-7 expression [144]. Decreased claudin-7 expression is also correlated with unfavorable prognostic factors, including invasion and lymph node metastasis, whereas patients with positive claudin-7 expression show a significantly favorable prognosis in oral squamous cell carcinoma [145].

The combined expression of several claudins can predict disease recurrence, as shown in a recent study by Lu *et al.* [13] in which the relationship between the expression of claudins -1, -3, -4, -7 and -8 and patient survival was analyzed in high-grade invasive breast cancer including several molecular subtypes. In this study, low expression of all five claudins was mostly detected in basal-like cancers (77%), and patients with claudin-low tumors had significantly shorter recurrence-free survival, suggesting that low levels of claudin expression predict disease recurrence.

An association between claudin-10 gene expression and disease recurrence in hepatocellular carcinoma was suggested by the finding that patients with high expression of claudin-10 had shorter disease-free survival [146]. Multivariate analysis confirmed that claudin-10 gene expression is an independent predictor in recurrence of hepatocellular carcinoma.

### 6.2. Claudins as Drug Targets in Cancer

Claudins have four transmembrane domains and two extracellular loops, and they are promising targets for antibody-based therapies. However, their hydrophobicity and low immunogenicity has made it difficult to raise antibodies targeting claudins. Antibodies that specifically recognize the extracellular loops of human claudin-3 [148] or claudin-4 [149] have been successfully produced, and the anti-claudin-4 antibody has shown therapeutic antitumor activity *in vitro* and *in vivo* [149]. Furthermore, a dual-targeting monoclonal antibody against claudin-3 and claudin-4 was recently prepared and shown to possess antitumor effects *in vitro* and *in vivo* [150], supporting the potential role of claudins as targets for therapeutic antibodies. This antibody successfully induced antibody-dependent cellular cytotoxicity (ADCC) and complement-dependent cytotoxicity (CDC) *in vitro* and inhibited tumor formation in SCID mice *in vivo*.

Claudin-3 and claudin-4 are receptors for CPE; therefore, the use of CPE to target claudins has been suggested. CPE treatment of ovarian cancer cells affects TJ function [75], and treatment with recombinant CPE fused to tumor necrosis factor has cytotoxic effects in ovarian cancer cells, supporting the possible development of CPE as targeted therapy for ovarian cancer [151]. Especially, CPE is effective in chemoresistant/recurrent ovarian cancer based on the high expression of claudin-3 and claudin-4 in chemoresistant/recurrent ovarian tumors [132].

On the other hand, studies have shown the therapeutic effects of siRNAs targeting claudin. *CLDN3* siRNA showed potent suppression of both tumor growth and metastasis in mouse and human ovarian tumor xenografts [95].

Taken together, the results of previous studies suggest that claudin-targeted drugs and therapies for cancer treatment are likely to be clinically applicable in the near future, although proof of concept for claudin-targeted therapy is not yet fully established.

## 7. Conclusions

The generally accepted concept that tumorigenesis is associated with loss of function of TJs implies that claudin expression is downregulated during tumor progression. However, claudin expression can be decreased or increased in human cancer in a tissue-specific manner, and a role for claudins in tumor progression has been suggested based on their effects on the migration and invasion of cancer cells and metastasis. Furthermore, claudin expression is significantly associated with patient survival or recurrence in some cancers, suggesting that these TJ proteins could be prognostic markers and promising therapeutic targets, although their exact role in cancer remains to be elucidated.

The recent identification of a claudin-low subtype of breast carcinoma characterized by enrichment of EMT markers and stem cell-like features has suggested a potentially important role for claudins in the acquisition of a CSC/TIC phenotype through EMT. Recent studies provided further evidence supporting the EMT-induced generation of breast CSC/TICs with claudin-low expression and the relevance of the claudin-low subtype in chemoresistance. In this context, a link between claudin, EMT, CSC/TICs and chemoresistance has been suggested. A critical issue in the current treatment of cancer is the association between the development of CSC/TICs and resistance to current chemotherapy and recurrence after initial treatment. Therefore, a better understanding of the role of claudins in EMT and CSC/TICs may provide important information to elucidate the molecular mechanisms of tumor recurrence and may help in the design of therapeutic strategies for chemoresistant and recurrent cancers.

## Figures and Tables

**Table 1:** Human claudin genes and protein information.

Gene	Chromosomal location	Transcript	Protein size (amino acids)	Molecular weight (Da)
*CLDN1*	3q28–q29	NM_021101.3	211	22,744
*CLDN2*	Xq22.3–q23	NM_020384.2	230	24,549
*CLDN3*	7q11.23	NM_001306.3	220	23,319
*CLDN4*	7q11.23	NM_001305.3	209	22,077
*CLDN5*	22q11.21	NM_001130861.1	218	23,147
		NM_003277.3		
*CLDN6*	16p13.3	NM_021195.4	220	23,292
*CLDN7*	17p13	NM_001307.4	211	22,390
			158	16,837
*CLDN8*	21q22.11	NM_199328.1	225	24,845
*CLDN9*	16p13.3	NM_020982.2	217	22,848
*CLDN10*	13q31–q34	NM_182848.2	226	24,251
		NM_006984.3	228	24,488
*CLDN11*	3q26.2–q26.3	NM_005602.5	207	21,993
*CLDN12*	7q21	NM_012129.2	244	27,110
*CLDN14*	21q22.3	NM_144492.1	239	25,699
		NM_012130.2		
*CLDN15*	7q11.22	NM_014343.1	228	24,356
*CLDN16*	3q28	NM_006580.2	305	33,836
*CLDN17*	21q22.11	NM_012131.1	224	24,603
*CLDN18*	3q22.3	NM_001002026.2	261	27,856
		NM_016369.3	261	27,720
*CLDN19*	1p34.2	NM_001123395.1	224	23,229
		NM_148960.2	211	22,077
*CLDN20*	6q25	NM_001001346.2	219	23,515
*CLDN21*	4q35.1	NA	NA	NA
*CLDN22*	4q35.1	NM_001111319.1	220	24,509
*CLDN23*	8p23.1	NM_194284.2	292	31,915
*CLDN24*	4q35.1	XM_001714660.1	205	22,802

The chromosomal location and transcript information (refseq number) of *CLDN* genes were obtained from the National Center for Biotechnology Information site (http://www.ncbi.nlm.nih.gov/). Information on the claudin proteins including protein size and molecular weight were obtained from Universal Protein Resource (UniProt; http://www.uniprot.org/). NA: not available.

**Table 2:** Claudin protein expression in human cancers.

Cancer	Claudin-1	Claudin-2	Claudin-3	Claudin-4	Claudin-7	Claudin-11
Breast	Down [39] (invasive breast carcinoma) Up [13] (basal type of high-grade invasive ductal breast carcinoma)	Down [42,43]	Up [44]	Up [44] Up [13,40] (basal-like type of high-grade invasive ductal breast carcinoma) Down [39] (invasive ductal breast carcinoma of grade 1)	Down [45] (invasive ductal breast carcinoma) Up [13] (luminal type of high-grade invasive ductal breast carcinoma)	
Cervical	Up [46]	Up [46]		Up [46]	Up [46]	
Colorectal	Up [47,48]		Up [47]	Up [47]		
Esophageal			Up [49]	Up [49]	Down [50]	
Gastric	Up [41]		Up [41]	Up [41,51]	Up [52]	Down [53]
Liver	Down [54]					
Melanoma	Up [55]					
Ovarian			Up [56–61] (normal: ovarian surface epithelium) Down [64] (normal: fallopian tube)	Up [56,57,60–62] Down [64] (normal: fallopian tube)	Up [63]	
Prostate	Down [65]	Down [42]	Up [66]	Up [66]	Down [65]	
Pancreatic				Up [67,68]		
Uterine			Up [69]	Up [69]		

**Table 3:** Roles of claudins in human cancer.

Claudins	Cancer	Function	*In vitro* or *in vivo*	Role	References
Claudin-1	Breast	Increase of cell migration	*In vitro*	Cancer promoting	[88]
	Breast	Anti-apoptotic effect	*In vitro*	Cancer promoting	[89]
	Colon	Increase of invasion and metastatic behavior	*In vitro* & *in vivo*	Cancer promoting	[48]
	Liver	Increase of invasion	*In vitro*	Cancer promoting	[90]
	Liver	Induction of EMT	*In vitro*	Cancer promoting	[91]
	Melanoma	Increase of cell motility and invasion	*In vitro*	Cancer promoting	[55]
	Oral	Increase of invasion	*In vitro*	Cancer promoting	[92]
	Gastric	Inhibition of tumorigenicity	*In vivo*	Tumor suppressive	[72]
	Lung	Inhibition of cell migration and invasion, *in vivo* metastasis	*In vitro* & *in vivo*	Tumor suppressive	[93]

Claudin-3	Ovarian	Increase of invasion	*In vitro*	Cancer promoting	[94]
	Ovarian	Promoting *in vivo* tumor growth and metastasis	*In vivo*	Cancer promoting	[95]
	Ovarian	Inhibition of *in vivo* tumor growth and metastasis	*In vitro* & *in vivo*	Tumor suppressive	[64]
	Ovarian	Suppression of EMT	*In vitro* & *in vivo*	Tumor suppressive	[96]

Claudin-4	Ovarian	Increase of invasion	*In vitro*	Cancer promoting	[94]
	Ovarian	Stimulation of angiogenesis	*In vitro* & *in vivo*	Cancer promoting	[97]
	Gastric	Inhibition of migration and invasion	*In vitro*	Tumor suppressive	[7]
	Ovarian	Suppression of EMT	*In vitro* & *in vivo*	Tumor suppressive	[96]
	Pancreatic	Suppression of cell invasion and metastasis	*In vitro* & *in vivo*	Tumor suppressive	[81]

Claudin-6	Gastric	Increase of proliferation, migration and invasion	*In vitro*	Cancer promoting	[98]
	Breast	Inhibition of anchorageindependent growth	*In vitro*	Tumor suppressive	[99]
	Breast	Inhibition of anchorageindependent growth, migration and invasion	*In vitro*	Tumor suppressive	[100]

Claudin-7	Colorectal	Increase of cell proliferation and tumorigenicity	*In vitro* & *in vivo*	Cancer promoting	[80]
	Ovarian	Increase of invasion	*In vitro*	Cancer promoting	[101]
	Esophageal	Decrease of cell growth and invasion	*In vitro*	Tumor suppressive	[84]
	Lung	Inhibition of migration and invasion, *in vivo* tumor growth	*In vitro* & *in vivo*	Tumor suppressive	[102]

Claudin-11	Bladder	Inhibition of cell invasion	*In vitro*	Tumor suppressive	[103]
	Gastric	Inhibition of cell invasion	*In vitro*	Tumor suppressive	[53]

**Table 4:** Clinical significance of claudin expression in human cancers.

Claudins	Cancer types	Association of claudin protein expression based on IHC and patient survival	Prognosis	Independent prognostic factor	Reference
Claudin-1	Breast	Low expression→shorter disease-free interval	Low→poor		[140]
	Colon	Low expression→shorter overall survival and disease-free survival	Low→poor	Independent predictor of recurrence and positive prognostic factor	[139]
	Colorectal	Low expression→shorter cancer-specific and disease-free survival	Low→poor		[109]
	Prostate	Low expression→shorter recurrence-free survival	Low→poor	Independent predictor of recurrence	[65]
Claudin-3	Breast	Positive expression→shorter disease-free survival (triple-negative) Positive expression→longer disease-free survival (luminal)	High→poor (triple-negative) High→better (luminal)		[141]
	Ovarian	High expression→shorter survival	High→poor	Independent negative prognostic factor	[105]
Claudin-4	Breast	High expression→shorter cancer-specific survival, overall survival and recurrence-free survival	High→poor	Independent negative prognostic factor	[142]
	Breast	Positive expression→longer disease-free survival (triple-negative) Positive expression→shorter disease-free survival (luminal type)	High→better (triple-negative) High→poor (luminal )		[141]
	Colorectal	Low expression shorter cancer-specific and disease-free survival	Low→poor		[109]
	Esophageal	Twist1 high/claudin-4 low expression→shorter overall survival	Twist1 high/claudin-4 low→poor	Independent positive prognostic factor	[107]
	Gastric	Moderate to strong staining→shorter overall survival	High→poor	Independent negative prognostic factor	[41]
	Gastric	High membranous expression→longer overall survival	High→better	Independent positive prognostic factor	[7]
	Pancreatic	Low gene expression *→shorter overall survival	Low→poor	Independent positive prognostic factor	[110]
Claudin-7	Breast	Positive expression→shorter recurrence-free survival	High→poor		[143]
	Lung	Low expression→shorter overall survival	Low→poor		[144]
	Oral	Positive expression→better prognosis	High→better		[145]
Claudin-10	Liver	High gene expression *→shorter disease-free survival	High→poor	Independent prognostic factor for disease recurrence	[146]

IHC: Immunohistochemistry.

* Claudin gene expression by qRT-PCR.
